# *KRE5* Suppression Induces Cell Wall Stress and Alternative ER Stress Response Required for Maintaining Cell Wall Integrity in *Candida glabrata*

**DOI:** 10.1371/journal.pone.0161371

**Published:** 2016-08-22

**Authors:** Yutaka Tanaka, Masato Sasaki, Fumie Ito, Toshio Aoyama, Michiyo Sato-Okamoto, Azusa Takahashi-Nakaguchi, Hiroji Chibana, Nobuyuki Shibata

**Affiliations:** 1 Department of Infection and Host Defense, Tohoku Medical and Pharmaceutical University, Sendai, Japan; 2 Department of Electronic and Information Engineering, Suzuka National College of Technology, Suzuka, Japan; 3 Medical Mycology Research Center, Chiba University, Chiba, Japan; Louisiana State University, UNITED STATES

## Abstract

The maintenance of cell wall integrity in fungi is required for normal cell growth, division, hyphae formation, and antifungal tolerance. We observed that endoplasmic reticulum stress regulated cell wall integrity in *Candida glabrata*, which possesses uniquely evolved mechanisms for unfolded protein response mechanisms. Tetracycline-mediated suppression of *KRE5*, which encodes a predicted UDP-glucose:glycoprotein glucosyltransferase localized in the endoplasmic reticulum, significantly increased cell wall chitin content and decreased cell wall β-1,6-glucan content. *KRE5* repression induced endoplasmic reticulum stress-related gene expression and MAP kinase pathway activation, including Slt2p and Hog1p phosphorylation, through the cell wall integrity signaling pathway. Moreover, the calcineurin pathway negatively regulated cell wall integrity, but not the reduction of β-1,6-glucan content. These results indicate that *KRE5* is required for maintaining both endoplasmic reticulum homeostasis and cell wall integrity, and that the calcineurin pathway acts as a regulator of chitin-glucan balance in the cell wall and as an alternative mediator of endoplasmic reticulum stress in *C*. *glabrata*.

## Introduction

Maintenance of cell wall integrity (CWI) is critical in fungal biology [1–6]. The fungal cell wall is required for normal cell growth and acts as a physical support for maintaining fungal cell shape and as a barrier for protection against harsh environments [7,8]. Changing environmental conditions induce various signal transduction pathways that contribute to remodeling of cell wall physiology [9]. Of these pathways, the CWI pathway depends on a signal transduction mechanism involving the MAP kinase cascade, whose triggers are directly involved in cell wall remodeling [10,11]. Some *in vitro* studies have shown that up-regulation of the CWI pathway in the pathogenic yeast *Candida glabrata* induce resistance to echinocandin antifungal drugs at clinically relevant supra-minimum inhibitory concentrations (MIC) [12–14]. It is also well known that lack of cell wall β-1,6-glucan causes severe growth defects and strongly induces CWI [15]. The cell wall of *C*. *glabrata* is composed of mannoproteins, β-1,3-glucans, β-1,6-glucans, and chitin [16]. Cell wall metabolism in *C*. *glabrata* has been characterized by comparative genomic analyses of *Saccharomyces cerevisiae* [17–19], and β-1,6-glucans in *C*. *glabrata* act as a linker between mannoproteins and chitin in the outer cell wall across the cell wall structure [15,20]. These findings suggest that β-1,6-glucans play an important role in maintaining a certain cell wall structure, and disruption of CWI is expected to be a new target for antifungal drugs.

A recent study showed that endoplasmic reticulum (ER) homeostasis is required for maintaining proper cell wall structure and for inducing antifungal resistance in many fungal species such as *Saccharomyces cerevisiae* [21], *Aspergillus fumigatus* [22], and *Cryptococcus neoformans* [23]. Unfolded protein response (UPR) is a well-conserved reaction in most eukaryotes for maintaining ER homeostasis [24,25]. *Saccharomyces cerevisiae* has a canonical UPR signaling system, the *IRE1-HAC1* pathway, whereas humans have two other UPR pathways [26–29]. *C*. *glabrata* lacks the canonical *IRE1*-*HAC1* pathway for the UPR, which is required for transmitting ER stress accumulation signals to the cytoplasm [30]. Nonetheless, *C*. *glabrata* has primary resistance against a typical ER stress inducer, tunicamycin (TM), and treatment with TM induces the expression of several genes required for maintaining the proper cell wall structure [31]. This suggests that *C*. *glabrata* has different UPR mechanisms regulating the CWI pathway.

*KRE5*, which belongs to the *KRE* family of genes, is predicted to be involved in cell wall β-1,6-glucan synthesis in many eukaryotes, including *Saccharomyces cerevisiae* [32] and *Candida albicans* [33]. *KRE5* encodes a soluble luminal ER protein containing a highly conserved UDP-glucose glycoprotein:glucosyltransferase (UGGT) domain in its *C*-terminus. In several eukaryotes, the UGGT domain is involved in the folding of nascent secretory proteins, including some cell wall component synthases, in the ER with the help of ER chaperones such as calnexin [34–36]. Deletion of the gene encoding UGGT induces dramatic cell wall alterations in many fungi [32,33,37]; however, the UGGT domain of *S*. *cerevisiae* Kre5p does not function as a co-chaperone of calnexin, in contrast to the Kre5p of other fungi [8]. Although *C*. *glabrata* is phylogenetically similar to *S*. *cerevisiae* [38], the function of *C*. *glabrata* Kre5p is unclear. Mutations in other *KRE* family genes result in a viable phenotype in most cases; however, mutations in *C*. *glabrata KRE5* induce a lethal phenotype described later in this study. Therefore, we hypothesized that *KRE5* has an epistatic function affecting the growth and CWI in *C*. *glabrat*a. In the present study, we characterized the functions of *C*. *glabrata KRE5* by generating a *KRE5* mutant with a regulatable gene expression system, and determined whether ER-mediated CWI was induced by the repression of ER-localized Kre5p.

## Results

### *C*. *glabrata* possesses a single gene similar to *Saccharomyces cerevisiae KRE5*

We identified an uncharacterized gene, CAGL0E05412g (http://www.genolevures.org/), that was highly similar to *Saccharomyces cerevisiae KRE5* (S1 Fig). CAGL0E05412g encoded a protein containing 1,326 amino acids, with a predicted molecular weight of 152.3 kDa and an ER translocation signaling sequence at its *N*-terminus. The *C*-terminus of the predicted protein contained a domain that was significantly similar to the UGGT domain, which is highly conserved in many eukaryotes and in the Kre5p of fungi belonging to the phyla Ascomycota. Therefore, the CAGL0E05412g gene was regarded as a functional homologue of *KRE5*, and was designated as *C*. *glabrata KRE5* (*CgKRE5*).

### *CgKRE5* is indispensable for cell survival

**Fig 1:**
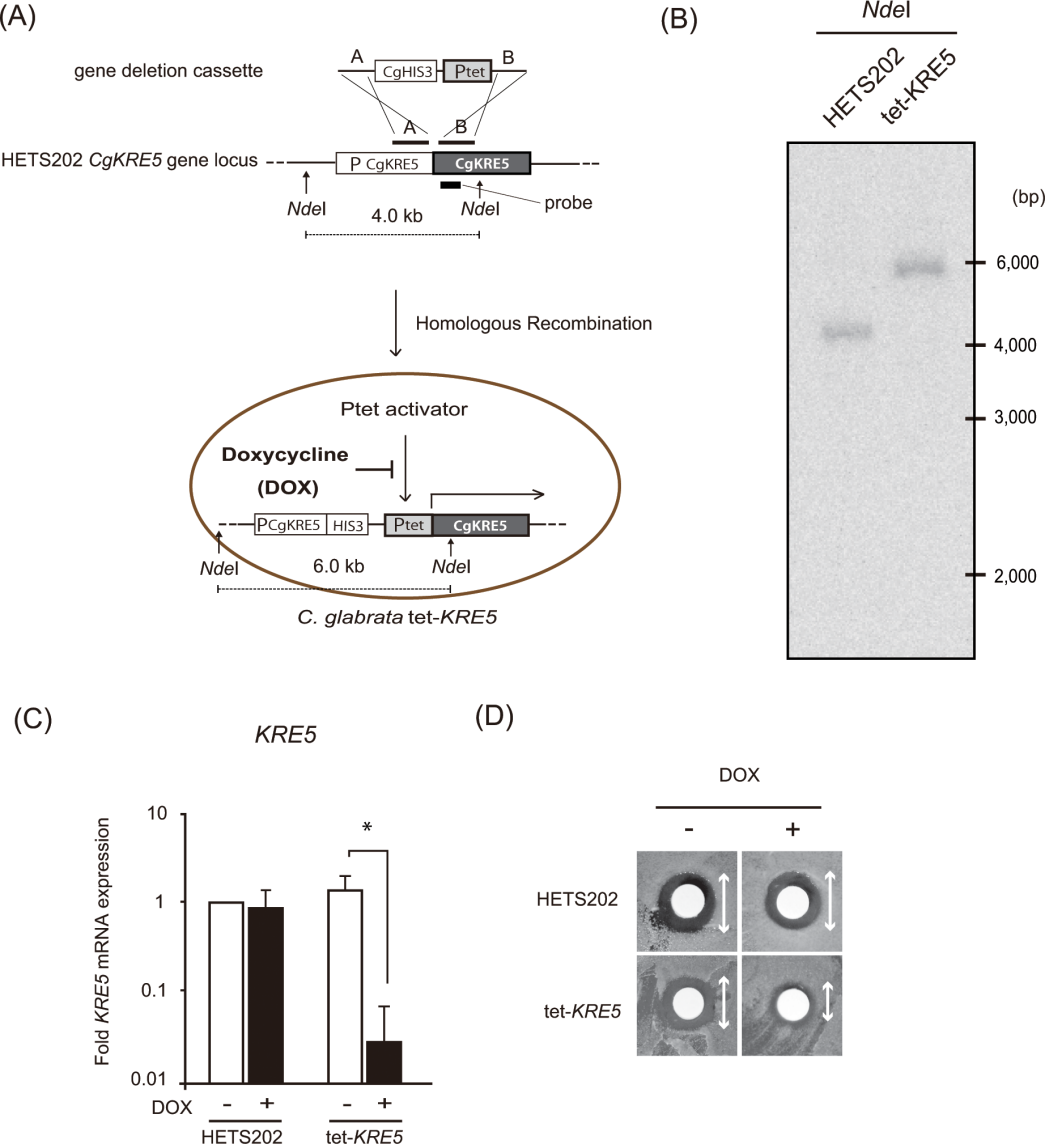
Generation of tet-*KRE5* cells. (A) Schematic representation of the integration of the controllable tet-off promoter. Region A represents the 5´ flanking region of the target gene and region B represents the 5´ end of the ORF. DNA fragment containing the controllable cassette in which *CgHIS3* and tetO-*ScHOP1* promoter were flanked by regions A and B, was used to transform *C*. *glabrata* HETS202 cells. (B) Correct integration of the tet-off promoter in the *CgKRE5* promoter locus was confirmed by Southern blot analysis. Genomic DNA isolated from both transformants and HETS202 cells was digested with *Nde*I, And hybridized by *CgKRE5* internal DNA probe. (C) Relative *CgKRE5* expression was determined by performing RT-PCR. Data are expressed as the mean fold difference between HETS202 and tet-*KRE5* cells treated with or without DOX. The value of HETS202 cells without DOX treatment was set as 1. Results are expressed as the mean standard deviation (S.D.) of triplicates; *, p < 0.05. (D) Inhibition ring test with K-1 killer toxin. *C*. *glabrata* cells were streaked on YPD solid medium, and 1 mg mL^-1^ of K-1 killer toxin solution was spotted onto paper discs that were then placed on the YPD solid medium inoculated with *C*. *glabrata*.

To investigate the role of *CgKRE5*, we replaced the *CgKRE5* ORF with a selectable marker; however, we could not generate a *CgKRE5* disruption mutant (data not shown). As reported previously, almost all haploid *KRE5*-null mutants of *Saccharomyces cerevisiae* strains are nonviable. Therefore, we predicted that *CgKRE5* disruption induced a lethal phenotype in *C*. *glabrata*. Then, we used a tetracycline-dependent system to repress *CgKRE5* (Fig 1A). A tetracycline-dependent down-regulatable promoter (tet-off promoter) [39] was inserted upstream of the *CgKRE5* ORF in the parent *C*. *glabrata* HETS202 strain, and the resulting strains were genotyped by Southern blot analysis to confirm the correct integration site (Fig 1B). The tet-off strain showed significant reduction in *CgKRE5* mRNA expression in the presence of 20 μg mL^−1^ doxycycline (DOX) (Fig 1C). Because mutations in *KRE* family genes induce a killer toxin-resistant phenotype in *Saccharomyces cerevisiae* [40], we performed an inhibition ring test to determine whether *CgKRE5* repression induced a killer toxin-resistant phenotype. We observed that DOX-treated cells incubated with K-1 killer toxin formed a small inhibition ring (Fig 1D). Furthermore, *CgKRE5* repression could be complemented by expression of *Saccharomyces cerevisiae* Kre5p (S2 Fig). Thus, we successfully generated a tet-regulatable *KRE5* strain with DOX-dependent *CgKRE5* expression, and *CgKRE5* was characterized as a *KRE* family gene even in *C*. *glabrata*.

### *CgKRE5* is involved in cell wall morphogenesis

**Fig 2:**
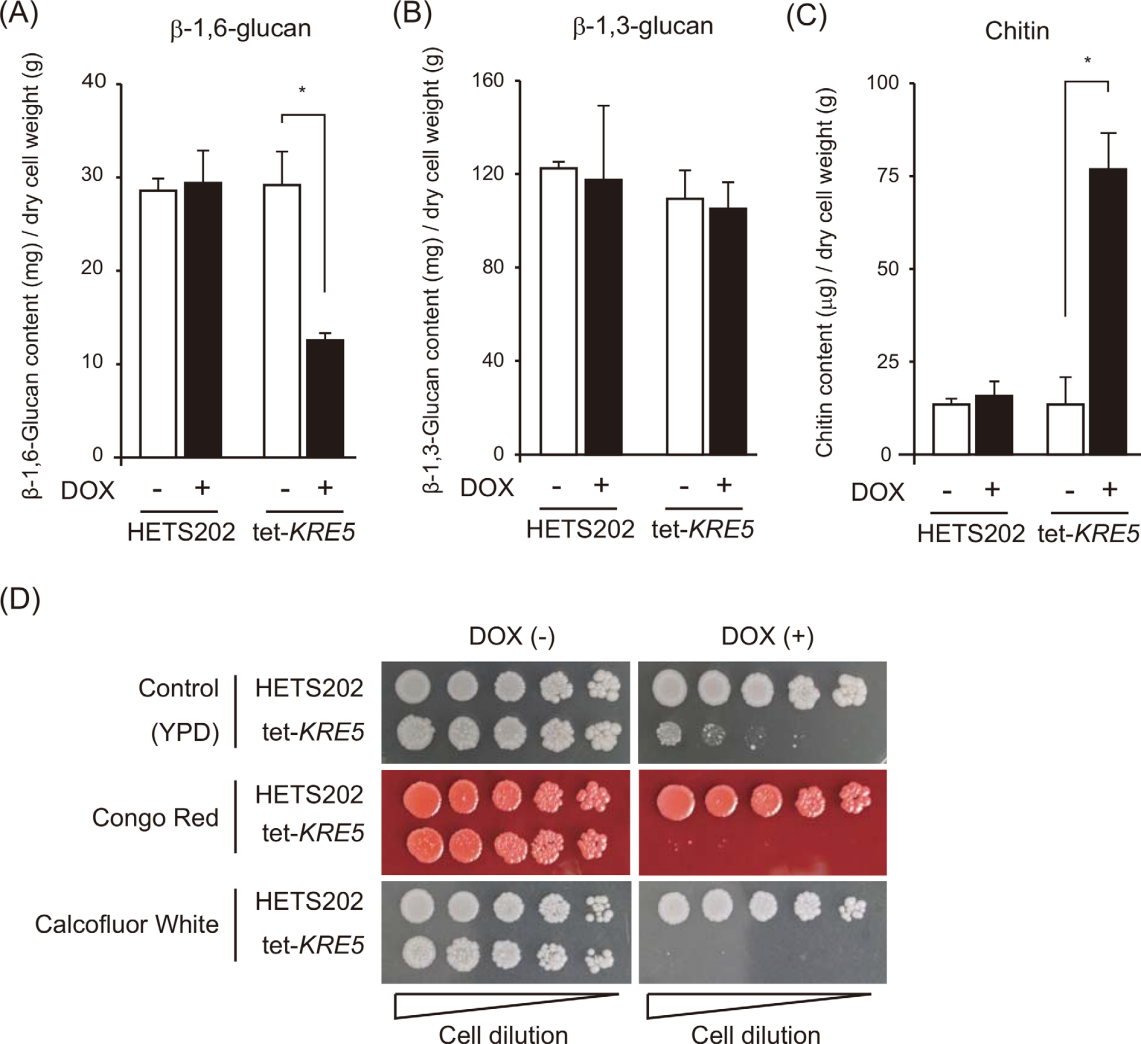
*CgKRE5* is involved in cell wall morphogenesis. Alkali-insoluble β-1,6-glucan (A), β-1,3-glucan (B), and chitin (C) contents in *C*. *glabrata* cells were measured as the quantity of glucose or glucosamine substituted for the standard curve. Data shown represent the results of at least three independent experiments. Error bars represent standard deviations; *, p < 0.05. (D) Spot dilution assay was performed using 5 μL suspensions at an OD of 0.1, and serially diluted (1:5) cells were spotted on yeast extract-peptone-dextrose (YPD) plates with or without DOX and were incubated at 37°C. A representative image of three independent experiments is shown.

Because KRE family genes are predicted to be involved in cell wall synthesis, we measured the major cell wall components, β-glucan and chitin, in the *CgKRE5* repression mutant. DOX-treated tet-*KRE5* cells showed approximately 50% reduction in β-1,6-glucan content (Fig 2A) but no significant reduction in β-1,3-glucan content (Fig 2B). This result indicates that *CgKRE5* plays an important role in maintaining cell wall β-1,6-glucan content. Furthermore, cell wall chitin content was considerably increased in DOX-treated tet-*KRE5* cells (Fig 2C). These data suggest that *CgKRE5* repression induced cell wall decomposition, which is characterized by a decrease in cell wall β-1,6-glucan content and an abnormal accumulation of cell wall chitin content. We next investigated whether *CgKRE5* repression affected not only cell wall structure but also cell wall homeostasis by effects on cell wall effector molecules that induce cell wall stress [41–43]. Compared to HETS202 cells and DOX-untreated cells, DOX-treated tet-*KRE5* cells showed a decreased rate of cell growth (Fig 2D). Importantly, DOX-treated tet-KRE5 cells exhibited hypersensitivity to Congo red (CR) and calcofluor white (CFW), which covalently bind to cell wall β-1,3-glucans and chitin, respectively (Fig 2D). These data imply that the DOX-treated tet-*KRE5* cells had a fragile cell wall because of the decrease in cell wall β-1,6-glucan content, and could not bear further stress. Moreover, these results indicate that appropriate *CgKRE5* expression was required for maintaining normal cell wall physiology, and that *CgKRE5* repression induced abnormalities in cell wall catabolism through the CWI pathway.

### *CgSLT2* mediates *CgKRE5* repression-induced cell wall chitin synthesis, and its deletion leads to further suppression of vegetative growth

**Fig 3:**
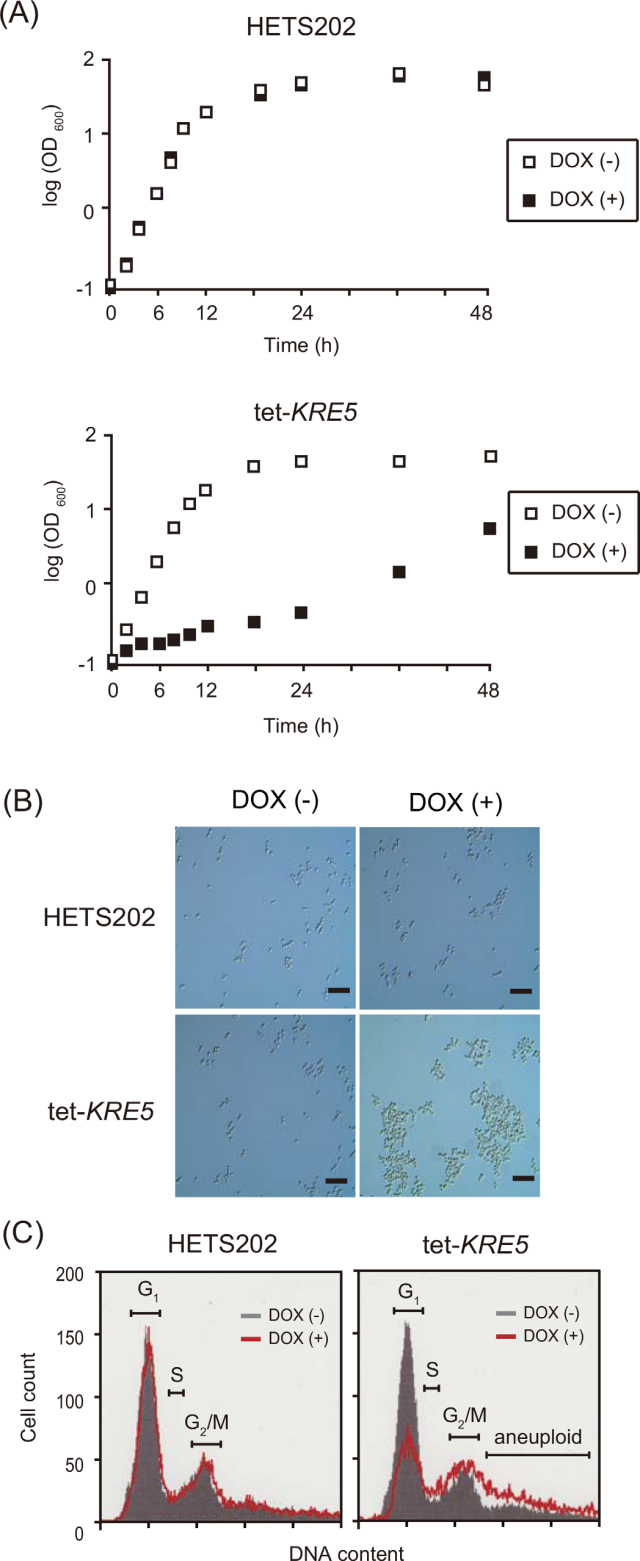
*CgKRE5* repression induces cell growth defect. (A) Growth curve of HETS202 and tet-*KRE5* cells. Cells were grown in YPD medium supplemented with or without DOX until they reached a stationary phase. Cell growth was recorded by examining aliquots of cell suspension at regular intervals. (B) Microscopic analysis of HETS202 or tet-*KRE5* cells. A representative image of three independent experiments is shown; scale bars: 10 μm (C) Flow cytometric analysis was performed using propidium iodide-stained HETS202 (left panel) and tet-KRE5 (right panel) cells treated with or without DOX. A representative image for three independent experiments is shown.

To determine the impact of these cell wall defects on cell viability, we evaluated important properties such as growth rate, cell shape, and cell cycle. DOX-treated tet-*KRE5* cells showed significant growth defects in liquid YPD medium (Fig 3A). This result indicates that *CgKRE5* is required for normal growth of *C*. *glabrata*. Microscopic analysis revealed that tet-*KRE5* cells were highly agglutinated, with an irregular shape and size (Fig 3B). Furthermore, the number of DOX-treated tet-KRE5 cells in the G_2_/M phase was lower than that of DOX-untreated cells (Fig 3C). In addition, the number of aneuploid cells in the DOX-treated tet-*KRE5* cells increased. These results suggest that Cg*KRE5* repression affected normal cell growth, which in turn exacerbated morphogenesis checkpoint defects and prevented proper bud formation as a result of the cell wall β-glucan-chitin imbalance.

**Fig 4:**
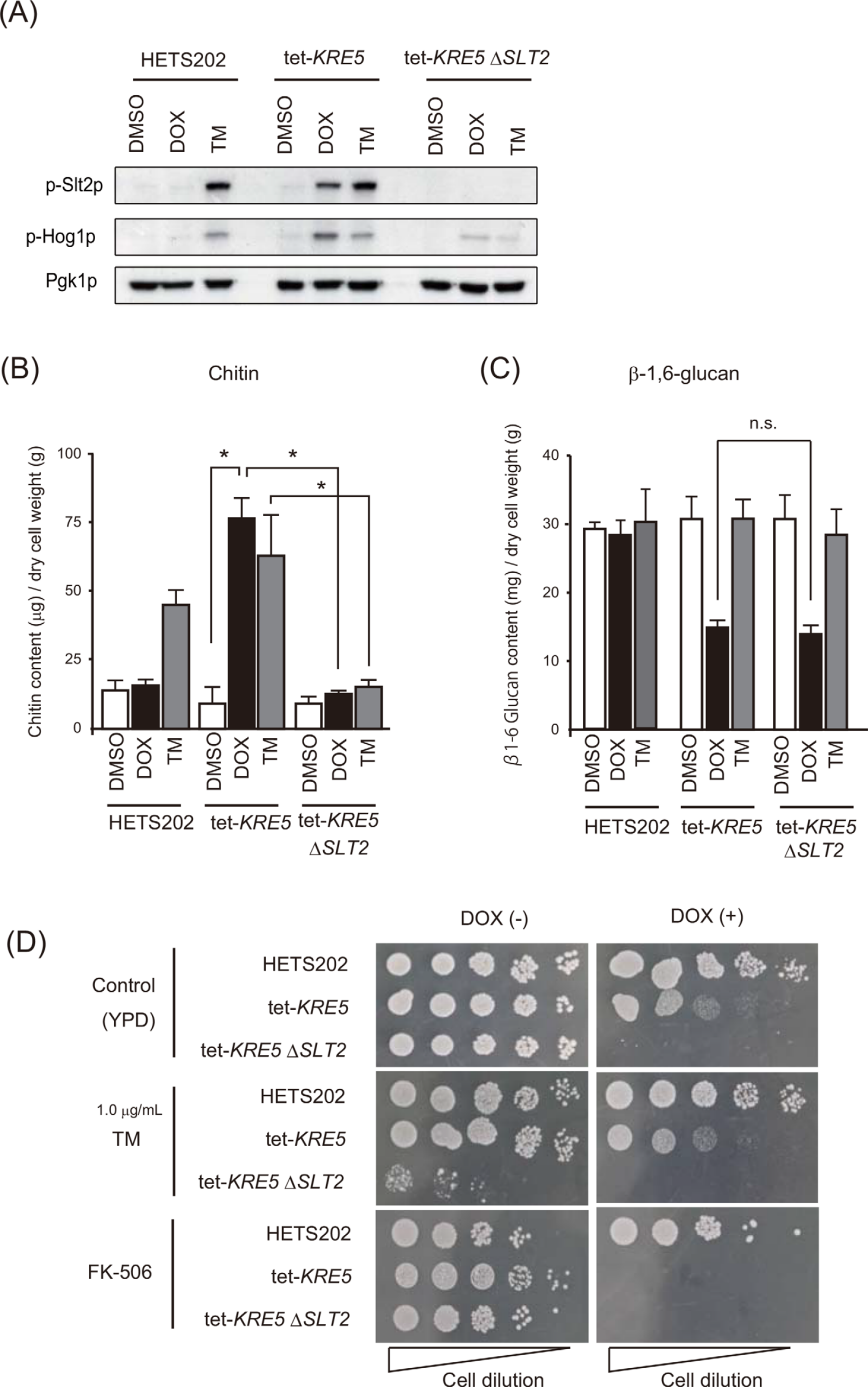
*CgKRE5* repression increased cell wall chitin content by activating the CWI-regulating MAP kinase pathway. (A) Phosphorylation of Slt2p and Hog1p was determined by performing western blotting analysis with antibodies against phosphorylated Slt2p and Hog1p (p-Slt2p and p-Hog1p, respectively). Anti-Pgk1p antibody was used as a loading control. Representative data of three independent experiments are shown; TM, tunicamycin. (B and C) Alkali-insoluble chitin (B) and β-1,6-glucan (C) content in *C*. *glabrata* cells were measured as the quality of glucose or glucosamine substituted for the standard curve. Data shown represent the results of at least three independent experiments. Error bars represent standard deviations; *, p < 0.05. (D) Spot dilution assay was performed using ER stress indicator. For this, 5 μL suspensions at an OD of 0.1 and serially diluted (1: 5) cells were spotted on YPD plates with the indicated concentration of reagents incubated at 37°C. A representative image of three independent experiments is shown.

Because up-regulation of chitin in the cell wall has been often observed in extrinsic cell wall damage, we considered that *CgKRE5* repression might activate CWI activation to compensate for the lethal cell wall defect. In the event of a cell wall defect, the intracellular *SLT2* and *HOG1* MAP kinase cascade is activated to maintain the cell wall structure [42,44]. Because Slt2p phosphorylation is required for the activation of the CWI pathway [45,46], we monitored the phosphorylation of both Slt2p and Hog1p to determine whether Cg*KRE5* repression causes CWI activation. Consistent with the cell wall alteration in *KRE5* repression, both Slt2p and Hog1p were significantly phosphorylated in DOX-treated tet-*KRE5* cells, suggesting that *CgKRE5* repression induces CWI pathway activation (Fig 4A). To determine whether Slt2p plays a role in cell wall chitin complementation in *CgKRE5* repression, we constructed a *CgSLT2* deletion mutant from the tet-*KRE5* strain. Combined disruption of *CgKRE5* and *CgSLT2* completely suppressed up-regulation of cell wall chitin (Fig 4B), but did not exert any effect on β-1,6-glucan content (Fig 4C). These data indicate that *CgKRE5* directly regulates β-1,6-glucan synthesis and indirectly induces chitin synthesis through the Slt2p pathway. Furthermore, *CgSLT2* deletion in the presence of *CgKRE5* repression significantly suppressed vegetative growth (Fig 4D, upper). These results suggest that Slt2-mediated CWI plays a crucial role in maintaining the cell wall under *CgKRE5* repression.

### *CgKRE5* repression induces ER stress

**Fig 5:**
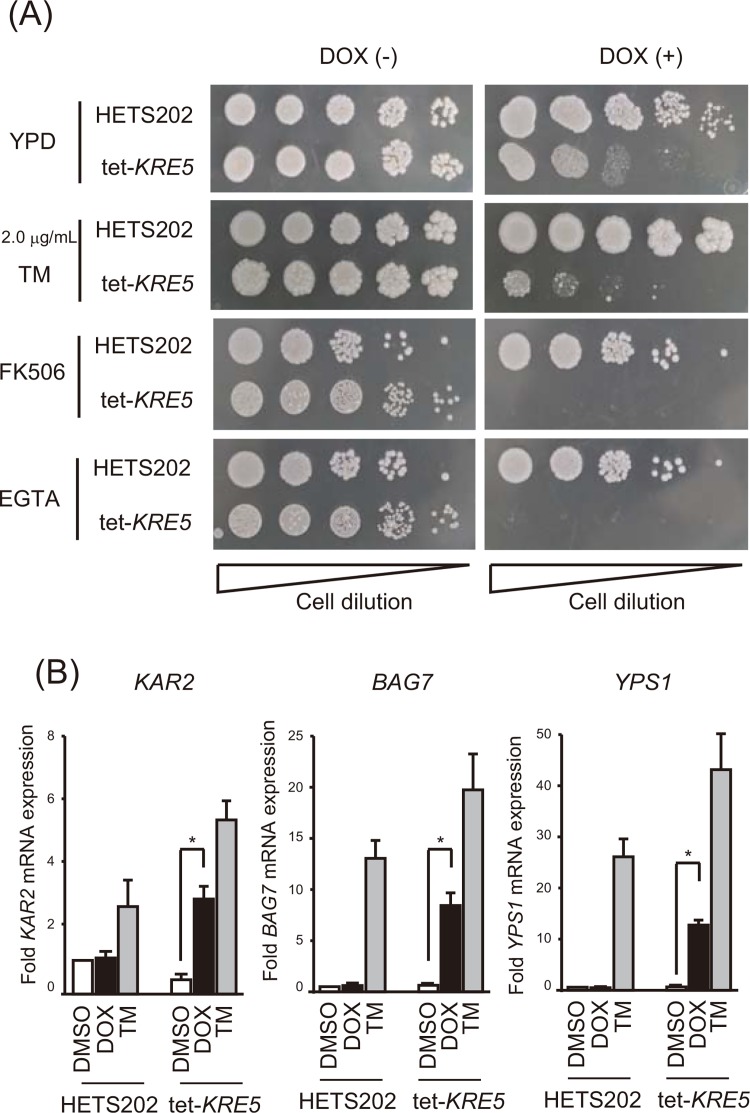
Repression of *CgKRE5* induces ER stress in *C*. *glabrata*. (A) Spot dilution assay was performed using the ER stress indicator. Five-microliter suspensions at an OD of 0.1 and serially diluted (1: 5) cells were spotted on YPD plates with the indicated concentration of reagents incubated at 37°C. A representative image of three independent experiments is shown. (B) Real-time RT-PCR was performed to measure the mRNA expression levels of ER stress-associated genes. Amplification efficiencies were validated and were normalized using that of *PGK1*. Relative mRNA levels were calculated as the ratio of normalized mRNA level to the mRNA level of *PGK1*. Values are represented as the average of three independent experiments, and error bars indicate S.D.; *, p < 0.05 compared with DMSO-treated tet-*KRE5* cells (Student´s *t*-test).

Next, we considered how *C*. *glabrata* senses cell wall abnormalities and transmits the signal for activating CWI by repressing the ER protein Kre5p. Treatment with the typical ER stress inducer TM resulted in phosphorylation of both Slt2p and Hog1p (Fig 4A). TM treatment increased cell wall chitin content in both HETS202 and tet-*KRE5* cells (Fig 4B), but did not affect β-1,6-glucan content (Fig 4C). Moreover, *CgSLT2* deletion in the presence of *CgKRE5* repression significantly suppressed growth with TM treatment (Fig 4D, middle). These data suggest that ER stress also activated Slt2p-mediated CWI in *C*. *glabrata*. In addition, we observed that the growth of TM-treated cells was comparable to that of the parental and DOX-treated cells (Fig 5A). These results led us to hypothesize that *CgKRE5* repression causes ER stress accumulation, and that ER stress induces CWI as UPR to dissolve structural abnormalities in the cell wall of *C*. *glabrata*. To confirm this, we initially analyzed the ER stress response after *CgKRE5* repression. Co-treatment of DOX-treated tet-*KRE5* cells with a calcineurin inhibitor FK-506 (tacrolimus) and Ca^2+^ chelator EGTA inhibited cell growth considerably (Fig 5A). Previous studies have suggested that the calcineurin pathway functions as the sole UPR pathway in *C*. *glabrata*, and that endogenous Ca^2+^ acts as an ER stress messenger [21,30,47]. Indeed, we observed that FK-506 treatment caused an extensive decrease of cell growth in DOX-treated tet-KRE5 and tet-KRE5*ΔSLT2* cells (Fig 4D, bottom). Consistently, our results suggest that *CgKRE5* repression induces hypersensitivity to exogenous ER stress induction and/or an imbalance in ER homeostasis, leading to the accumulation of endogenous basal ER stress. Moreover, recent studies have indicated that deletion of the ER stress-related protein, Ire1p, activates the CWI pathway. Therefore, we believe that *CgKRE5* repression induces other cellular reactions, such ER stress and/or UPR, which lead to abnormal cell growth. To further determine whether *CgKRE5* repression induced ER stress in *C*. *glabrata*, we examined the mRNA expression levels of representative UPR target genes, including *KAR2* (a resident ER chaperone), *BAG7* (a putative GTPase-activating protein involved in cell wall and cytoskeleton homeostasis), and *YPS1* (involved in ER protein trafficking) via real-time PCR. Expression of these UPR target genes increased in DOX-treated tet-*KRE5* cells (Fig 5B) and in TM-treated cells compared with that in DOX-treated cells, indicating that *CgKRE5* repression increased ER stress in *C*. *glabrata*.

### *CgKRE5* repression-induced up-regulation of cell wall chitin content is further enhanced by treatment with the calcineurin inhibitor FK-506

**Fig 6:**
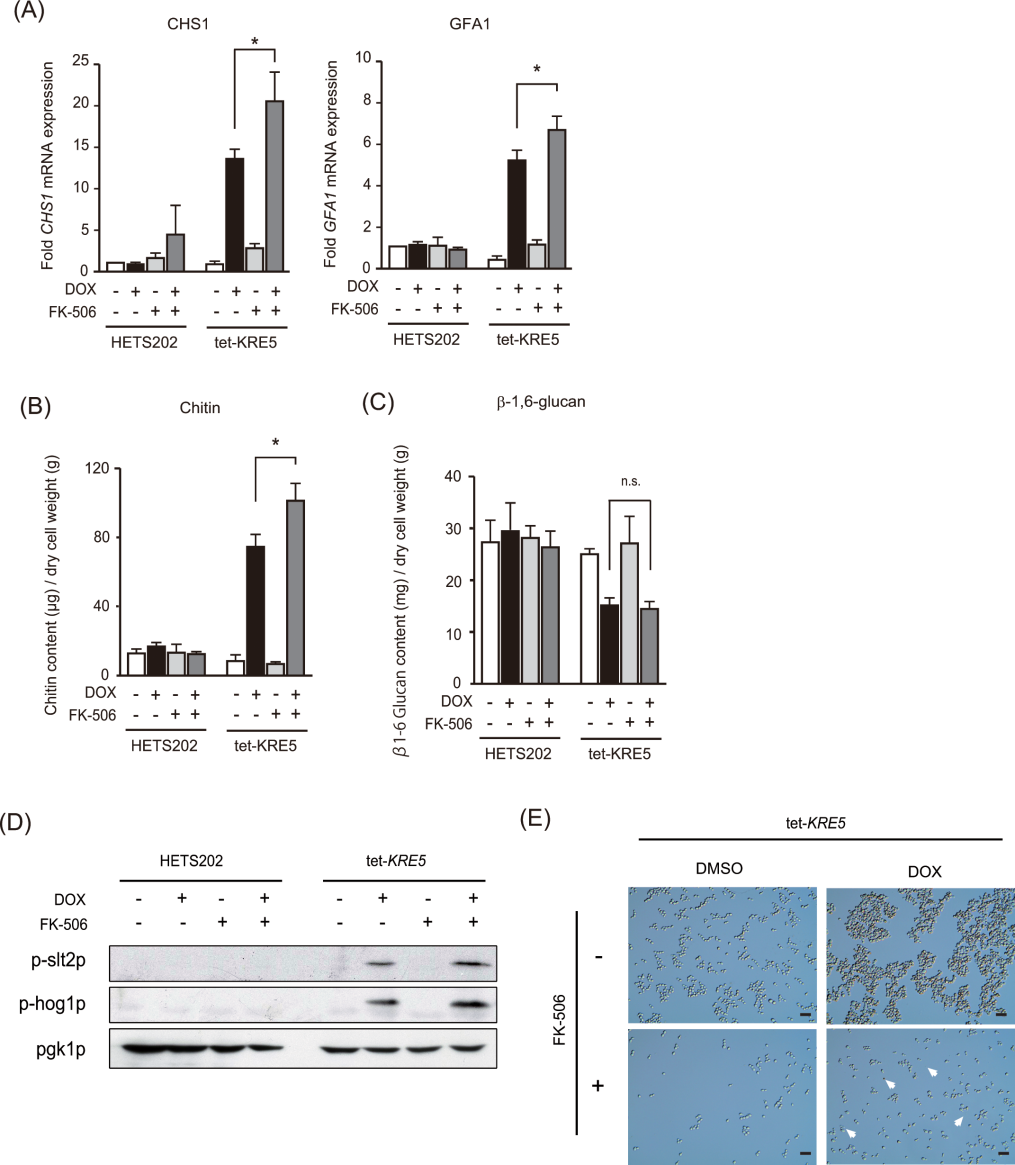
Calcineurin inhibitor FK-506 upregulated cell wall chitin synthesis by accelerating Slt2p phosphorylation. (A) Relative mRNA levels of *CHS1* and *GFA1* were calculated from the ratio of the signal intensities of *GFA1* and *CHS1* mRNAs compared with that of *PGK1* mRNA, which was used as a reference. Values are represented as an average of three independent experiments, and error bars indicate S.D.; *, p < 0.05, compared with DOX-untreated tet-*KRE5* cells (Student´s *t*-test). (B and C) Cell wall composition of *C*. *glabrata* strains is shown. Alkali-insoluble chitin (B) and β-1, 6-glucan (C) contents in *C*. *glabrata* cells were measured as the quantity of glucosamine or glucose substituted for the standard curve. Data shown represent the results of at least three independent experiments. Error bars represent standard deviations; *, p < 0.05; n.s, not significant. (D) Phosphorylation of Slt2p and Hog1p was determined by performing western blotting analysis with antibodies against p-Slt2p and p-Hog1p, respectively. Anti-Pgk1p antibody was used as the loading control. Representative data are shown of independent experiments is shown. (E) Microscopic analysis of tet-*KRE5* cells. A representative image of three independent experiments is shown; scale bar: 10 μm; arrow, disrupted *C*. *glabrata* cells.

As mentioned previously, *CgKRE5* repression induced both cell wall decomposition and ER stress by phosphorylating Slt2p MAP kinase in *C*. *glabrata*. To investigate whether inhibition of *CgKRE5* repression-induced ER stress affected cell wall structure, we co-treated tet-*KRE5* cells with DOX and FK-506. Co-treatment of tet-KRE5 cells with DOX and FK-506 increased the transcriptional activation of *CHS1* and *GFA1* compared with that in tet-*KRE5* cells treated with DOX alone (Fig 6A). *CHS1* encodes chitin synthase, which is required for forming primary chitinous septal plate [48], and *GFA1* encodes glutamine:fructose-6-phosphate amidotransferase, which is required for the synthesizing metabolic precursors of cell wall chitin [49]. Increased *CHS1* and *GFA1* mRNA expression in tet-KRE5 cells co-treated with DOX and FK-506 increased cell wall chitin content (Fig 6B), but did not significantly affect cell wall β-1,6-glucan content (Fig 6C). Similarly, Miyazaki *et al*. reported that TM treatment induced *CHS1* and *GFA1* mRNA expression in *C*. *glabrata* [47], suggesting that *CgKRE5* repression induced cell wall chitin content by activating transcription of *CHS1* and *GFA1* as an ER stress response. Moreover, it was suggested that the calcineurin pathway negatively regulated cell wall chitin synthesis.

To determine whether calcineurin regulated the Slt2p MAP kinase pathway, we analyzed Slt2p phosphorylation in FK-506-treated tet-*KRE5* cells. Co-treatment of tet-*KRE5* cells with FK-506 and DOX increased phosphorylation for both Slt2p and the Hog1p, whereas treatment of tet-*KRE5* cells with only FK-506 did not affect the phosphorylation of either Slt2p or Hog1p (Fig 6D). Moreover, *CgSLT2* deletion did not rescue the vegetative growth of FK-506-treated cells (Fig 4D). These data imply that ER stress activates the calcineurin pathway and partly regulates CWI driven by Slt2p activation. Remarkably, FK-506 treatment rescued cell aggregation induced by *CgKRE5* repression but resulted in an increased number of ruptured cells (Fig 6E). These data suggest that inhibition of the calcineurin pathway induces cell growth defects under cell wall stress, and that the calcineurin complex plays an important role in negatively regulating the Slt2p pathway in CWI in *C*. *glabrata*.

### *CgKRE5* repression enhances the sensitivity of *C*. *glabrata* to micafungin

**Fig 7:**
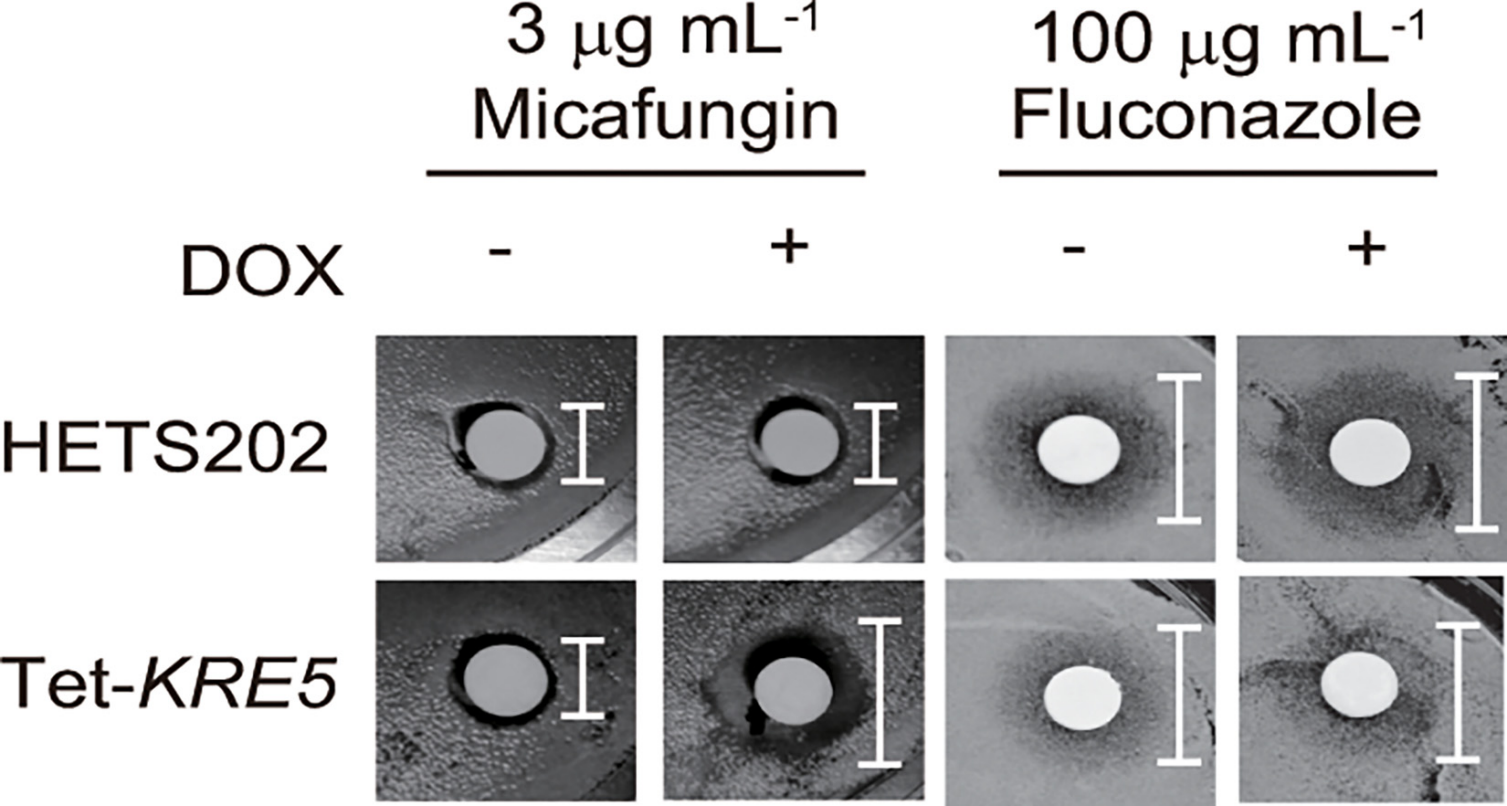
*CgKRE5* repression enhances the sensitivity of *C*. *glabrata* to micafungin. Inhibition ring test by using micafungin or fluconazole is shown. *C*. *glabrata* cells were streaked on YPD solid medium, and indicated concentrations of micafungin or fluconazole were spotted onto paper discs, which were then placed on the YPD solid medium inoculated with *C*. *glabrata*.

The integrity of cell surface components, including the cell wall and cell membrane, affects the efficacy of antifungal drugs. Recent studies have shown that increased cell wall chitin content thorough activation of the Slt2p induces resistance to echinocandin drugs in some *Candida* spp., including *C*. *glabrata*. Therefore, we examined the sensitivity of tet-*KRE5* cells to micafungin and fluconazole. No significant difference in fluconazole sensitivity was observed after DOX treatment; however, DOX-treated tet-*KRE5* cells showed modestly impaired growth in the presence of micafungin compared to the DOX-untreated cells (Fig 7). This result indicates that *CgKRE5* repression enhances the sensitivity of *C*. *glabrata* to micafungin, despite the increase in cell wall chitin content.

## Discussion

### *CgKRE5* plays an important role in the biosynthesis of β-1,6-glucan and maintenance of ER homeostasis

Cell wall β-1,6-glucan biosynthesis is one of the most intriguing processes in fungal cell biology. The *KRE* family gene products are localized in the ER-to-Golgi secretory pathway and are believed to be a key component for cell wall β-1,6-glucan synthesis [50]. Although it is still unclear which enzyme directly catalyzes this process and where the β-1, 6-glucan is synthesized, our data clearly indicate that repression of Cg*KRE5* induced ER stress accumulation and dynamic cell wall decomposition, with a higher reduction in cell wall β-1,6-glucan content. *CgKRE5* dysfunction induces cell wall stress by impairing the balance of cell wall β-glucan content and increased basal ER stress levels. In many eukaryotes, glycoprotein synthesis through the ER-to-Golgi secretory pathway is globally down-regulated under excessive ER stress accumulation [51]. The ER-to-Golgi secretory pathway promotes β-1,6-glucan synthesis, suggesting that important glycoproteins involved in β-1,6-glucan synthesis are down-regulated under ER stress after *CgKRE5* repression. Kurita *et al*. reported that proper folding and localization of Kre6p and Knh1p and of Cwh41p and Rot2p, which are localized in the ER and which act as co-chaperones of calnexin (Cne1p), are required for β-1,6-glucan synthesis [52]. In addition, a recent study showed that loss of the UPR function induces cell wall defects and that cell wall stress activates the UPR through the MAP kinase signaling pathway regulating CWI [53]. Consistent with these results, we found that loss of ER homeostasis induced defects in cell wall β-1,6-glucan biosynthesis and activated the CWI pathway.

However, the actual functions of *CgKRE5* are still unclear. *Schizosaccharomyces pombe gpt1* encodes a protein containing the UGGT domain [37] with an α-1,3-glucosyl transferase activity that adds a glucose residue to the terminal mannose of an immature *N*-linked glycan in the ER. In addition, a recent study showed that Gpt1p is involved in the UPR along with Ire1p, a luminal sensor of misfolded ER proteins in *Schizosaccharomyces pombe* [54] However, *Saccharomyces cerevisiae* Kre5p does not show such activity [8]. *C*. *glabrata* Kre5p contained the UGGT domain and a predicted ER retention signaling sequence at its C-terminus. We observed that *CgKRE5* repression induced ER stress accumulation, which indicates that *CgKRE5* is required for maintaining ER homeostasis in *C*. *glabrata*, similar to *Schizosaccharomyces pombe gpt1*.

### Increased cell wall chitin content does not induce resistance to echinocandin drugs

Echinocandins such as micafungin and caspofungin inhibit the synthesis of cell wall β-glucans by competitively inhibiting β-1,3-glucan synthase Fks1p in *C*. *glabrata*. Previous studies have suggested that echinocandin treatment of most *Candida* spp. at supra-MIC induces paradoxical growth improvement by increasing cell wall chitin content and *SLT2* mRNA expression [55–58]. In contrast, our results suggest that *CgKRE5* repression induced micafungin hypersensitivity, despite activation of cell wall chitin synthesis and increased cell wall chitin content in *C*. *glabrata*. Echinocandin antifungal drugs inhibited β-1,3-glucan synthesis, and *CgKRE5* repression decreased β-1,6-glucan content, implying that compensatory activation of chitin synthesis does not reverse excessive β-1,6-glucan synthesis in *C*. *glabrata*, and possibly in other fungi, similar to that observed with the CWI pathway. Although β-1,6-glucan content is much lower than β-1,3-glucan content in the cell walls of *Candida* spp., β-1,6-glucan chains play a pivotal role in the assembly of cell wall components [59,60]. The results of previous studies and those of the present study suggest that resistance to echinocandin antifungal drugs is not induced by an increase in cell wall chitin content. Overall, it is clear from our findings that inhibition of the β-1,6-glucan synthesis is a promising antifungal target.

### Calcineurin inhibitor FK-506 disrupts fungal CWI, indicating its use as a potential antifungal drug

Calcineurin, a Ca^2+^/calmodulin-dependent phosphatase complex, has been investigated in detail mainly in *S*. *cerevisiae* and in mammalian cells. Previous studies have demonstrated that the calcineurin pathway plays pivotal roles in *C*. *glabrata* biology, such as in cell wall maintenance, antifungal susceptibility, and virulence induction in mice and other eukaryotes [61,62]. Recently, Miyazaki *et al*. reported that TM-induced ER stress results in the transcriptional activation of genes involved in chitin synthesis and that calcineurin and the Slt2p MAP kinase cascade play a uniquely important role in the ER stress response in *C*. *glabrata*, rather than the canonical UPR mechanism, i.e., the *IRE1*-*HAC1* pathway [31]. We observed that *CgKRE5* repression induced ER stress accumulation, Slt2p phosphorylation, and *CHS1* and *GFA1* mRNA up-regulation, thus increasing cell wall chitin content. Furthermore, inhibition of the calcineurin pathway activated the CWI pathway of cell wall remodeling and induced greater impairment of fungal cell growth. These findings indicate that the calcineurin pathway negatively regulates cell wall chitin content similarly to the CWI pathway under cell wall stress. In many fungi, the balance between cell wall chitin and β-glucan content is essential for normal cell growth. Therefore, calcineurin plays an important role in maintaining CWI by negatively regulating ER stress-mediated chitin synthesis.

We observed that the calcineurin inhibitor FK-506 effectively disrupted the cell wall β-glucan-chitin balance under cell wall stress conditions. The defect in β-1,6-glucan synthesis induced by *CgKRE5* repression was not dependent on the ER stress response, at least in the calcineurin pathway. A recent *in vitro* study showed that co-treatment of *C*. *glabrata* with tacrolimus and azole antifungal drugs exerted a strong antifungal effect [63], suggesting that inhibition of the calcineurin pathway induces severe growth defects in *C*. *glabrata* under cell wall stress, which was consistent with the findings of the present study. Patients with acute graft-versus-host disease (GvHD), a life-threatening immunological complication that occurs after hematopoietic stem cell transplantation, are treated with tacrolimus (FK-506) or cyclosporine A [64,65]. These immunosuppressive drugs are currently administered as prophylactic agents not only for preventing GvHD but also for preventing fungal infection after solid-organ transplantation [66,67]. Consistent with the application in these clinical therapies, our results suggest that FK-506 induced an imbalance in cell wall β-glucan and chitin content, thus inducing cell growth defects in *C*. *glabrata*, and that it exerted potential antifungal effects by disrupting fungal CWI.

Previous studies reported synergic effects of tacrolimus and azole antifungal agents on inhibition of the synthesis of fungal-type cell membranes[68,69]. In our experiment, fluconazole, a representative azole antifungal drug, did not exert antifungal activity under repression of *KRE5*. This result indicates that *KRE5* repression-induced CWI maintained not only the cell wall but also the cell membrane, i.e., the cell “surface”. Therefore, co-treatment with tacrolimus and an azole antifungal drug might be more efficient against *C*. *glabrata* infection because tacrolimus would function as a CWI disruptor under cell wall stress conditions.

### Concluding remarks

**Fig 8:**
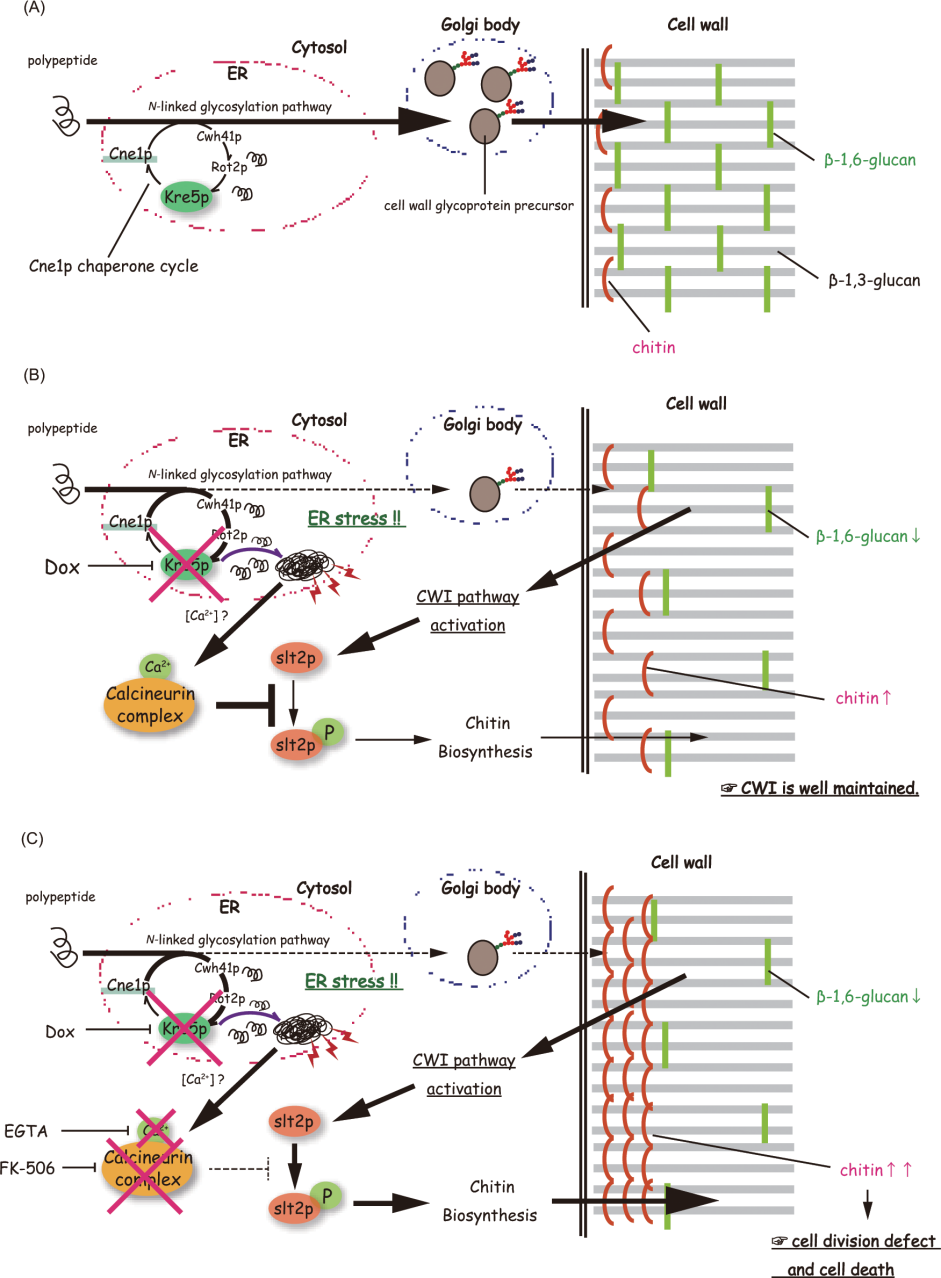
A model showing the roles of the CWI and calcineurin pathway in response to Cg*KRE5* repression. (A) During normal cell growth, Kre5p serves as a chaperone cycle member for the proper folding of *N*-linked glycoprotein required for the cell wall synthesis in ER. (B) *CgKRE5* repression induces CWI and UPR. The calcineurin pathway acts as a coordinating role for maintaining an appropriate chitin content in *CgKRE5* repression-mediated CWI. (C) FK-506 prevents the calcineurin pathway and induces an imbalance in cell wall structure.

We clarified that *CgKRE5* regulated CWI in *C*. *glabrata*. Our results also suggest that *CgKRE5* plays a pivotal role in the maintenance of CWI because *CgKRE5* dysfunction induced ER stress and activated the CWI pathway, which led us to propose a hypothetical model (Fig 8). During normal cell growth, delivery of cell wall precursors through the intracellular ER-to-Golgi secretory pathway maintains an appropriate cell wall structure. In the ER, Kre5p serves as a chaperone cycle member for proper folding of *N*-linked glycoproteins, which is a probable step in the synthesis of cell wall β-1,6-glucan (Fig 8A). *CgKRE5* repression considerably decreases cell wall β-1,6-glucan content and the reciprocal increases cell wall chitin content by activating the CWI pathway. At the same time, Kre5p repression in *C*. *glabrata* induces ER stress and also the sole UPR in *C*. *glabrata*, the calcineurin pathway. Thus, CWI is maintained by maintenance of an appropriate chitin content by the calcineurin complex through an “intracellular check” (Fig 8B). FK-506 prevents the formation of the calcineurin complex, thus repressing the negative regulation of the CWI pathway. Increased cell wall chitin content resulting from dysfunction of the calcineurin pathway induces an imbalance in cell wall structure and affects normal cell growth (Fig 8C). Our findings support recent arguments that the UPR activates the CWI pathway in some pathogenic fungi. To the best of our knowledge, this is the first study to report that inhibition of the ER stress pathway exerts harmful effects on *C*. *glabrata* under cell wall stress.

## Experimental Procedures

### Strains and culture conditions

The *C*. *glabrata* strains used in this study are listed in S1 Table. A transactivator-expressing HETS202 strain was were used to generate tet-*KRE5* strains [70]. *C*. *glabrata* strains were grown at 37°C on a YPD complex medium containing 2% glucose, 2% peptone (Kyokuto, Japan), and 1% yeast extract (Nacalai Tesque, Japan). YPD agar plates were prepared by adding 2% agar (Nacalai Tesque) to the YPD medium. A yeast nitrogen base (0.67% YNB [Difco Laboratories, United States]) containing 2% glucose and 2% agar with appropriate amino acids and bases was used as a selective medium after HETS202 cells were transformed. Yeast transformations were performed using a modified lithium acetate method [71].

### Generation of conditional mutants

Transformation of *C*. *glabrata* was performed as previously described [70]. Primers for strain construction were designed using Primer3Plus (http://www.bioinformatics.nl/cgi-bin/primer3plus) and are listed in S2 Table. HETS202 cells were transformed using a DNA cassette prepared by PCR with primers p12874F and p12874TR for tet-*KRE5* and with plasmid pTK916-97t as a template. Insertion of the DNA cassette upstream of the *KRE5* ORFs in transformed cells was verified by colony PCR with primer p22F and p12874tcheck and Southern blot analysis.

The Tet-*KRE5ΔSLT2* strain was constructed from the *tet-KRE5* strain. Approximately 1.0 kb of DNA from the 5´ and 3´UTRs flanking the *CgSLT2* coding sequence was amplified by PCR with the genomic DNA of *C*. *glabrata* as a template and with primers CgSLT2 5Fw and CgSLT2 5Rv + hphMX, and CgSLT2 3Rv + hphMX and CgSLT2 3Rv. A gene replacement cassette was constructed by performing fusion PCR with each PCR fragment and *hphMX* with primers CgSLT2 3rdFw and CgSLT2 3rdRv. Homologous recombination in the transformants was confirmed by PCR with primers CgSLT2 5Fw and CgSLT2 3Rv.

### Sequence analysis

CAGL0E5412 and *ScKRE5* were aligned using T-COFFEE version 11.00 (http://tcoffee.crg.cat) and were visualized using ESPrint 3.0 (http://espript.ibcp.fr/ESPript/ESPript/).

### Southern blotting analysis

Southern blot analysis was performed following a standard protocol [72]. Genomic DNAs isolated from HETS202 and tet-*KRE5* strain were digested overnight with *Nde*I. Digested DNAs were separated in 0.8% agarose gel in 1×TAE buffer, and transferred to a Hybond-N^+^ membrane (GE Healthcare, United States). A PCR-amplified DNA fragment of *CgKRE5* was used as a probe (the primes used are listed in S2 Table). DNA probes were randomly labeled with [α-^32^P] dCTP (PerkinElmer, United States) by using the Random Primer DNA labeling kit ver.2 (Takara, Japan). Membranes were prehybridized for 1 h in 50 mL of 20× SSC solution and hybridized overnight at 65°C for the ^32^P-labeled probes. After hybridization, the membrane was rinsed twice with 2× SSC- 0.1% SDS solution at 65°C. The hybridized membrane was visualized in a BAS-5000 system (Fuji film, Japan). Raw data of the analysis are shown in S4 Fig.

### Drug susceptibility assay

Drug susceptibility was determined using spot dilution test. Cells were cultured in liquid YPD medium at 37°C until they reached an exponential phase, and their density was adjusted to approximately 2 × 10^8^ cells mL^-1^. Next, 5 μL drops of serially diluted (1:5) cell suspension were spotted onto YPD agar plates containing the following: DOX (20 μg mL^-1^, Sigma-Aldrich, United States), CFW (600 μg mL^-1^; Sigma-Aldrich), CR (600 μg mL^-1^; Sigma-Aldrich), TM (1.5 μg mL^-1^; Nacalai Tesque), and FK-506 (100 ng mL^-1^; Nacalai Tesque). The cells were cultured at 37°C for 48 h, and cell growth was observed.

### Flow cytometry for cell cycle analysis

Flow cytometry for cell cycle analysis was conducted as described previously by Borah *et al*. [73], with few modifications. Logarithmically growing cells were harvested, washed twice with water, and fixed in 1 mL of ice-cold 70% ethanol at 4°C for 16 h. The fixed cells were washed with 1 mL of 50 mM sodium citrate buffer, suspended in 0.5 mL of 50 mM sodium citrate buffer containing 0.1 mg mL^-1^ RNase A (Nippon Gene, Japan), and incubated at 37°C for 2 h. Propidium iodide (Nacalai Tesque) was added at a final concentration of 50 ng mL^-1^. The cells were sonicated for 30 s to prevent doublet formation and were analyzed by flow cytometry with a FACSCalibur system (BD Biosciences, United States) at an excitation wavelength of 488 nm. A minimum of 20,000 events was recorded for each sample, and data were analyzed using CellQuest Pro software (BD Biosciences).

### Preparation of alkali-insoluble fraction and analysis of cell wall composition

Cells were cultured in liquid YPD medium in the presence or absence of 20 μg mL^-1^ DOX at 37°C until they reached the exponential phase. Next, the cells were washed 3 times with deionized water, collected by centrifugation, and extracted using 1% NaOH at 100°C for 24 h. The pellet obtained was washed twice with deionized water and extracted using 0.5 M acetic acid at 80°C for 24 h. The pellet was washed twice with deionized water and lyophilized.

The alkali-insoluble β-glucan content was determined as described by Umeyama *et al*. with some modifications. Briefly, 1.0 mg of lyophilized alkali-insoluble fraction was suspended in 10 mM Tris-HCl (pH 7.4) containing 1.0 mg mL^-1^ Zymolyase-100T (Nacalai Tesque), and incubated at 37°C for 24 h. The precipitate obtained was removed by centrifugation at 15,500 × g for 10 min, and half of the supernatant was dialyzed overnight against 10 mM Tris-HCl (pH 7.4). The hexose content was determined using the phenol H_2_SO_4_ method.

The total cell wall chitin content was determined as described by François with some modifications [74]. Briefly, 20 mg of lyophilized alkali-insoluble fraction was hydrolyzed using 1 M H_2_SO_4_ at 100°C for 4 h and neutralized. The precipitate obtained was removed by centrifugation, and the supernatant obtained was dissolved in 2 mL of deionized water after evaporation to dryness. Next, 500 μL of the solution was added to 1 mL of acetylacetone solution (10% [v/v] acetylacetone in 1.25 M sodium carbonate) and incubated at 90°C for 1 h, followed by addition of 10 mL of 100% ethanol and 1 mL of Reissig reagent (1% 4-dimethylaminobenzaldehyde, 1.25% [v/v] HCl in glacial acetic acid). The chitin content was determined by measuring absorbance of glucosamine at 490 nm.

### mRNA extraction and reverse transcription-PCR

Logarithmic-phase cells (OD_600_ = 0.1) were inoculated in YPD medium supplemented with or without the required chemicals and were grown at 37°C for 4 h. For reverse transcription-PCR, total RNA was isolated using Sepazol RNA I Super G (Nacalai Tesque) according to the manufacturer’s instructions. The isolated RNA was treated with DNase I to remove any residual DNA, and 500 ng of total RNA was reverse transcribed to cDNA by using ReverTra Ace qPCR Master mix (Toyobo, Japan). Next, 1 μL of the resulting RT reaction mixture was used as a template for performing individual PCR with Thunderbird SYBR qPCR mix (Toyobo). Real-time PCR was performed in triplicate in a 96-well plate by using a StepOnePlus Real-Time PCR System (Applied Biosystems, United States). Relative expression ratios were calculated using the ΔΔCt method. *PGK1* was used as a normalization reference for determining target gene expression level, and DMSO-treated HETS 202 cells were used as calibrators in each experiment. Primers for real-time PCR were designed using Primer3Plus and are listed in S2 Table. The assays were repeated at least twice independently.

### Western blotting analysis

Logarithmic-phase cells were inoculated in YPD medium and incubated at 37°C for 4 h. Next, the cells were washed twice with ice-cold deionized H_2_O and suspended in 200 μL of homogenizing buffer (50 mM Tris [pH 7.5] and 1 mM EDTA) containing 1× PhosSTOP (Roche, Switzerland) and 1× Complete (EDTA-free; Roche). The cells were then lysed by using glass beads and vortexing at the maximum speed. Cell debris and unbroken cells were removed by centrifugation at 13,000 ×g and 4°C for 10 min. Protein content was quantified using a XL-Bradford protein assay kit (APRO Science, Japan) per the supplier’s instructions. Next, 40 μg of the total protein was resolved by SDS-PAGE on a 10% gel and transferred onto PVDF membranes (PALL Life Science) that were then blocked using Blocking One-P (Nacalai Tesque) for 1 h at room temperature. Immunoblotting was conducted using an anti-phosphorylated-p44/42 MAP kinase antibody (#4370; Cell Signaling Technology, United States) and anti-Pgk1p antibody (Abcam, United Kingdom) at a dilution of 1:10,000 in TBS and 0.1% Tween 20 for 1 h at room temperature. HRP-linked anti-rabbit IgG (Promega, United States) was used as the secondary antibody and the blots were developed using an ECL plus western blotting detection system (GE Healthcare). The assays were repeated at least twice independently. The raw data of each analysis are shown in S5 Fig.

### Microscopy

Logarithmic-phase cells (OD_600_ of 0.1) were inoculated in YPD medium supplemented with or without the required chemicals and grown at 37°C for 4 h. The cells were harvested, washed twice with water, and fixed using 1 mL of 4% paraformaldehyde in phosphate-buffered saline at 4°C for 16 h. The cells were then embedded in Permafluor (Thermo scientific, United States) on a slide glass and observed under a differential interference contrast microscope (BX-53; Olympus; Japan). All images were acquired under identical conditions and processed in parallel.

## Supporting Information

S1 Fig Sequence analysis of *CgKRE5*. (EPS)

S2 Fig Complementation of *CgKRE5* knockdown/knockout by expressing ScKre5p. (A) Homologous recombination with *CgKRE5* gene disruption cassette was performed under ScKre5p expressing mutant. A ScKre5p expression vector pGRB2.2-ScKRE5 or an empty vector pGRB2.2 was transformed in *C*. *glabrata* strain NAU3. *CgKRE5* gene disruption cassette was then transformed in these strains and observed whether transformants were obtained. (B) Spot dilution assay was performed using ScKre5p expression tet-*KRE5* mutant, tet-*KRE5ΔURA3* pGRB2.2-*ScKRE5*. Empty vector pGRB2.2 was also transformed in tet-*KRE5ΔURA3* (tet-*KRE5ΔURA3* pGRB2.2). Five-microliter suspensions at an OD of 0.1 and serially diluted (1: 5) cells were spotted on SC URA^-^ plates with the indicated concentration of reagents incubated at 37°C.
(PDF)

S3 Fig Knock out Confirmation of *SLT2* gene in tet-*KRE5* strain. (EPS)

S4 Fig Original blots data for Southern blot analysis. (EPS)

S5 Fig Original blots data for western blot analysis. (EPS)

S1 Table Strain list used in this study. (XLSX)

S2 Table Primer list used in this study. (XLSX)

## Abbreviations

MAP kinase: mitogen-activated-protein kinase
