# Supplementary figures and images for: KRE5 Suppression Induces Cell Wall Stress and Alternative ER Stress Response Required for Maintaining Cell Wall Integrity in Candida glabrata
Source: PLoS One. 2016 Aug 22;11(8):e0161371. doi: 10.1371/journal.pone.0161371 (PMC4993462; doi:10.1371/journal.pone.0161371)

(A)

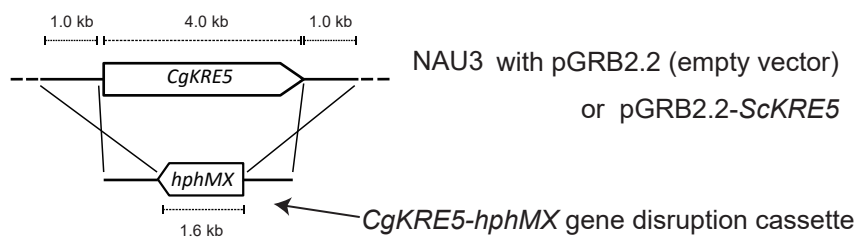

|                     | NAU3-pGRB2.2 |     | NAU3-pGRB2.2- <i>ScKRE5</i> |     |
|---------------------|--------------|-----|-----------------------------|-----|
| disruption cassette | (-)          | (+) | (-)                         | (+) |

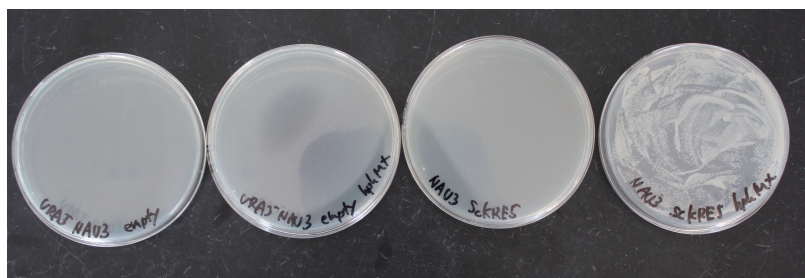

(B)

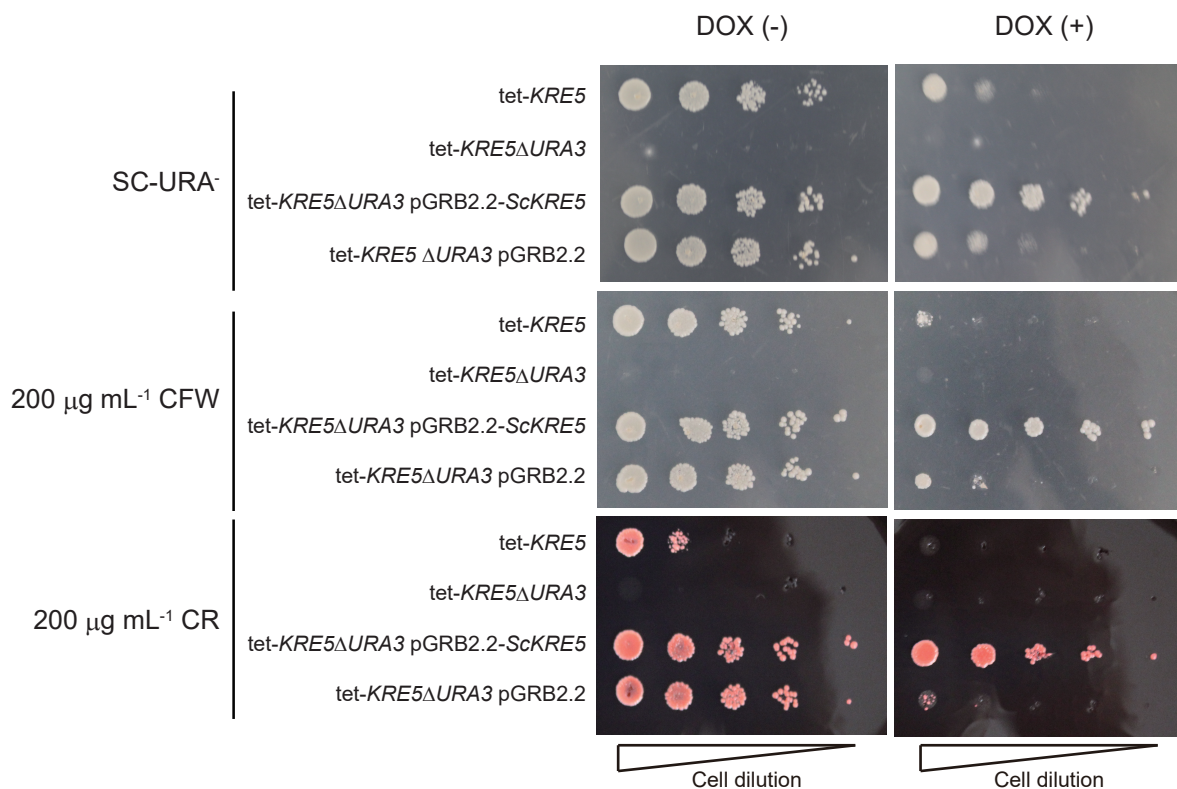

Supplement: S2 Fig — (A) Homologous recombination with CgKRE5 gene disruption cassette was performed under ScKre5p expressing mutant. A ScKre5p expression vector pGRB2.2-ScKRE5 or an empty vector pGRB2.2 was transformed in C. glabrata strain NAU3. CgKRE5 gene disruption cassette was then transformed in these strains and observed whether transformants were obtained. (B) Spot dilution assay was performed using ScKre5p expression tet-KRE5 mutant, tet-KRE5ΔURA3 pGRB2.2-ScKRE5. Empty vector pGRB2.2 was also transformed in tet-KRE5ΔURA3 (tet-KRE5ΔURA3 pGRB2.2). Five-microliter suspensions at an OD of 0.1 and serially diluted (1: 5) cells were spotted on SC URA- plates with the indicated concentration of reagents incubated at 37°C. (PDF) [file pone.0161371.s002.pdf]
